# Law of Mass Action Type Chemical Mechanisms for Modeling Autocatalysis and Hypercycles: Their Role in the Evolutionary Race

**DOI:** 10.1002/cphc.202000355

**Published:** 2020-07-10

**Authors:** Attila K. Horváth

**Affiliations:** ^1^ Department of Inorganic Chemistry Faculty of Sciences University of Pécs Ifjúság u. 6. H-7624 Pécs Hungary

**Keywords:** autocatalysis, landolt reaction, nonlinear dynamics, reaction mechanism, supercatalysis

## Abstract

One of our most appealing challenge is to unravel the role of a presumably autocatalytic system in controlling the origin and spreading of Life on our entire planet. Here we show that in the simplest autocatalytic loop involving reactions capable of self‐replication and obeying law of mass action kinetics, concentration growth of the autocatalyst may be characterized by parametrization of direct and autocatalytic pathways rather than by kinetic orders of the autocatalyst. Extending this model by feasible elementary steps allows us to outline super‐exponential growth where kinetic order of the autocatalyst is higher than unity. Furthermore, it is shown in case of the simplest hypercycle that such a situation might appear where the otherwise more sluggish autocatalytic route receives a decisive support from the crosscatalytic pathway to become an apparently stronger autocatalytic loop even if the other route contains a more efficient autocatalysis. If the hypercycle is performed under flow conditions selection of autocatalyst depends on kinetic and flow parameters influenced by external factors mimicking that the most adaptive loop of hypercycle eventually finds its wining way in the evolutionary race.

## Graphical abstract

**Law of mass action** type kinetic models are established to characterize the sim‐ plest hypercycle containing autocatalytic loops under batch and flow conditions. It is shown that the most adaptive autocatalytic route to the given external cir‐ cumstances in a hypercycle finally paves its winning way in the evolutionary race.

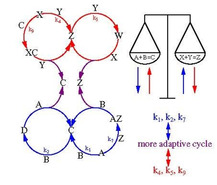

## 1 Introduction

Studying birth, emergence and spreading of life on our planet is being considered as furnishing challenges to generations of intuitive researchers who are curiously engaged in hunting the Holy Grail of the origin of Life in the prehistoric Earth. Debate on whether the ‘replication first’ or the ‘metabolism first’ theory is responsible for the origin of life is still an open question and possibly it seems to be standing for quite a while.^1^ Substantial difference between these schools of thought is that ‘replication first’ theory prefers that oligomeric compounds capable of self‐replication had to be first formed^2^ while in case of the other theory emergence of cyclic networks such as hypercycles^3^ had to be initially evolved. Hypercycle – in general ‐ is defined by coupling at least two autocatalytic reactions which are mutually catalytic for the autocatalyst of the other reaction cycle involved in the main loop. Soon after this idea was introduced in the 70s it met a serious criticism by an evolutionary biologist Smith, that hypercycles may be considered as unstable entities and they are unable to evolve characteristics without the favor of growth of the *per se* closed‐cycle itself.^4^ Therefore some forms of compartmentalization were also required to keep these hypercycles working in reality.^5^ Though the definition seems to be unambiguous, sometimes coupled cyclic biochemical systems without the inclusion of replicators or autocatalysis are misidentified^6^, ^7^ as hypercycles noticed by Szathmáry.^8^ Additional examples may as well be mentioned from nowadays reports^9^, ^10^, ^11^ indicating that this misconception may be more widespread than one would otherwise expect.^12^

Even though the approaches mentioned above seem to be different at a first sight it is generally accepted that autocatalysis must have played a crucial role in both possibilities meaning that these schools of thought may stem from a common core.^13^ The importance of autocatalytic reaction networks in prebiotic chemistry has been thoroughly analyzed by Blackmond.^14^ It was unambiguously shown that only truly autocatalytic cycles exhibit the critical characteristics to provide persistence to a prebiotic reaction cycle.

A reaction is called autocatalytic when one of its product enhances the rate of its own formation. This process is generally described in a simple form as

(1) A+B→C

along with its empirical rate equation

(2) $$\upsilon =k{}_{0}\left[A\right]\left[B\right]+k{}_{a}\left[A\right]\left[B\right]\left[C\right]{}^{P}$$

where *p*>0 is defined as the kinetic order of autocatalyst. In 1993 von Kiedrowski claimed that basically there are only two autocatalytic reaction orders for describing a self‐replicating system, when *p* is considered to be unity (case A) or *p*=0.5 (case B).^15^ This idea was introduced as a consequence of his previous work when template‐directed condensation of two trinucleotides leading to the formation a hexameric template with palindromic sequence was reported.^16^ In case A the growth of template molecule is considered to be exponential while in case B it is called parabolic. This nomenclature is also used in a recent review where the mechanisms of autocatalysis were surveyed.^17^ It is, however, interesting to note that although the term ‘mechanism’ was consistently mentioned in that paper, in kinetic sense a ‘mechanism’ has to consist of elementary or quasi‐elementary steps exclusively, where law of mass action type kinetics is uniquely fulfilled for each and every step. Misuse of term ‘mechanism’ and ‘kinetic model’ is not restricted to self‐replication studies it may as well be found in other fields^18^, ^19^, ^20^ thus the problem seems to be more general than one would otherwise expect. Recently, Virgo *et al*. showed that in case of a polymerization model not only first‐order autocatalysis but even more complex overall positive feedback processes may appear resulting in a superexponential growth.^21^ The model they used seems to satisfy the criterion of using law of mass action type kinetics although the data presented in their tables are shown in an unconventional way indicating the rate of reactions at a certain time instance without inferring the corresponding rate coefficients and rate laws.

Involvement of autocatalysis may further be highlighted by the following examples related to biological systems. Ibarz and Augusto have shown that autocatalytic kinetic model is capable of describing microbial growth during fermentation.^22^ Furthermore, Reuveni *et al*. demonstrated that ribosomes are functionally optimized for their autocatalytic production.^23^ It is also interesting to mention that a couple of years ago Bissette *et al*. reported the possibility of micelle‐mediated physical autocatalysis in a thiol–ene reaction^24^ followed by a subsequent study to analyze the variety of novel applications of physical autocatalysis.^25^

The aim of this study is to shed light on the critical role of autocatalysis modeled by the sequence of law of mass action type reactions and to emphasize that in batch conditions the growth of autocatalyst concentration may rather be characterized by parametrization of the direct pathway and the autocatalytic loop. We also show that in case of more complex systems, like hypercycles under batch conditions, where at least two autocatalytic reactions are interlinked by mutually catalyzing each other, an otherwise more sluggish autocatalytic pathway may receive a very efficient support from the catalytic route to become more active than the other autocatalytic route which has a more wholesome autocatalytic pathway without any significant assistance from its cross‐catalytic route. Furthermore, when a hypercycle is performed under flow conditions the selection among competitive autocatalysts depends on the kinetic and flow parameters influenced by external factors such as rate coefficients, temperature, concentration fluctuations, inflow and outflow rates, etc. suggesting that the best adaptive cycle finally finds its decisive role in the evolutionary race.

## 2 Results and Discussion

### 2.1 The Simplest Law of Mass Action Type Autocatalytic Mechanism

Let us consider reactants A and B producing C by the following sequence of reactions along with their corresponding rate laws all obeying law of mass action type kinetics:

(3) $$A+B\overset{k_{1}}{\to }C,\;v_{1}=k_{1}\left[A\right]\left[B\right]$$

(4) $$B+C\overset{k_{2}}{\to }D,\;v_{2}=k_{2}\left[B\right]\left[C\right]$$

(5) $$A+D\overset{k_{3}}{\to }2C,\;v_{3}=k_{3}\left[A\right]\left[D\right]$$

**Figure 1:**
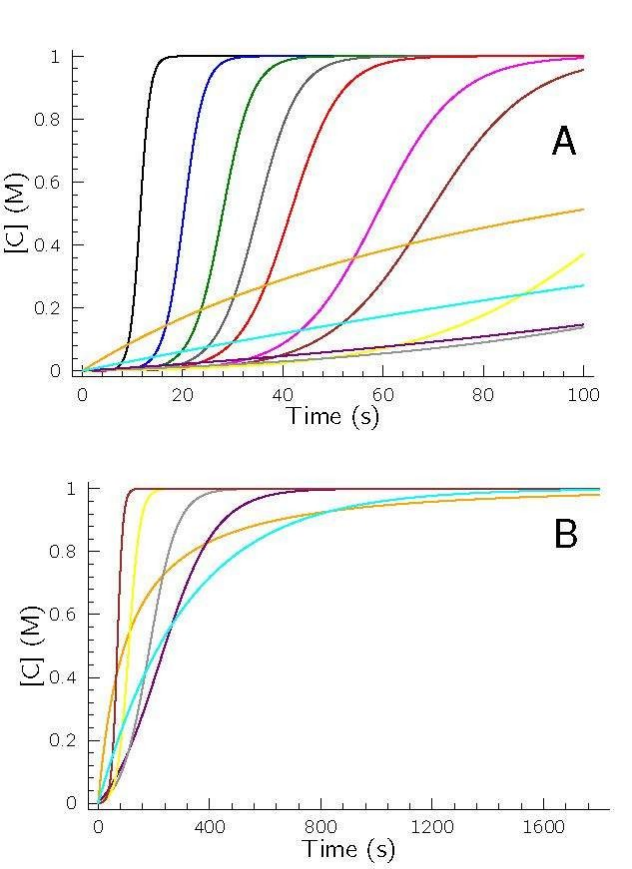
Concentration–time profiles of the autocatalyst at constant *k*_1_ *k*_2_=10^−5^ M^−2^s^−2^ value when varying the ratio of these rate coefficients. [A]_0_=[B]_0_=1.0 M, [C]_0_=0 M and *k*_3_=10^7^ M^−1^ s^−1^ was fixed. (A) *k*_2_ */k*_1_=10^5^ (black), 2.5×10^4^ (blue), 1.111×10^4^ (green), 6250 (dark grey), 4000 (red), 1562.5 (magenta), 1000 (brown), 250 (yellow), 40 (light grey), 10 (purple), 1 (cyan), 0.1 (orange). (B) At lower *k*_2_ */k*_1_ values the time scale was extended in order to reach at least 95 % conversion in case of smaller *k*_2_ */k*_1_ values.

Let us also suppose that eq. 5 is much faster than eqs. 3 and 4 thus *k*_3_≫*k*_2_ and *k*_3_≫*k*_1_ inequalities are fulfilled and the reaction is performed at batch conditions. It is easy to realize that sum of eqs. 4 and 5 gives the overall reaction represented by eq. 3, thus sequence of eqs. 4 and 5 forms an autocatalytic cycle (having a stoichiometry to be the same as that of eq. 3) because the rate law of the rate determining step in this sequence is proportional to the concentration of product C. It should also be noted that the kinetics of several real chemical systems may easily be described by this core mechanism.^26^, ^27^, ^28^, ^29^, ^30^ Among them the most well‐known is the bisulfite–iodate (Landolt) reaction discovered more than a century ago^31^ and, as a result, this sequence of reactions is called Landolt‐type systems.^32^ Figure 1 displays the concentration–time profiles of the autocatalyst at different *k*_2_ */k*_1_ ratios meanwhile keeping the product of *k*_1_ and *k*_2_ constant at an arbitrarily chosen 10^−5^ M^−2^ s^−2^ value.

**Figure 2:**
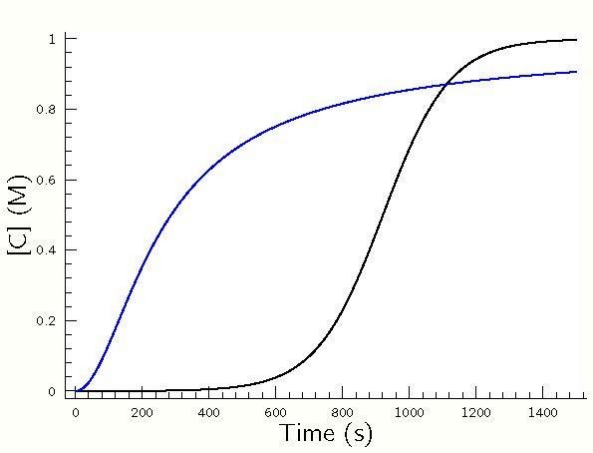
Simulated concentration–time profiles using eqs. 3–5 with *k*_1_=10^−6^ M^−1^ s^−1^, *k*_2_=10^−2^ M^−1^ s^−1^ and *k*_3_=10^7^ M^−1^ s^−1^ in case of the black curve as well as using eqs. 1–2 with *k*_0_=10^−13^ M^−1^ s^−1^ and *k_a_*=8×10^−3^ M^−1.5^ s^−1^ in case of the blue curve.

As it is shown, at higher *k*_2_ */k*_1_ values – meaning that the rate coefficient of the autocatalytic route is significantly higher than that of the nonautocatalytic pathway –, the concentration–time profiles display characteristic sigmoidal‐shaped curves suggesting the possible emergence of autocatalysis. At the highest value, concentration of the autocatalyst increases suddenly reaching its final value within a relatively short period of time at the given conditions. If, however, *k*_2_ */k*_1_ decreases meaning that the nonautocatalytic pathway starts to compete with the autocatalytic route, growth of [C] becomes less intense, though the sigmoidal‐shaped concentration–time profiles are still preserved. A more intense increase in the concentration of autocatalyst in such systems is generally called ‘exponential’ growth, while the opposite one refers to ‘parabolic’ increase and interpreted in terms of the formal kinetic orders of the autocatalyst being one and half, respectively.^15^, ^17^, ^33^ Our simulations here, however, suggest that the exponential and parabolic growth should be the consequence of parametrization rather than that of different kinetic order of the autocatalyst being involved in the kinetic model. As a result, differentiation and interpretation of these situations must necessarily rest on the ratio of rate coefficients assigned for the autocatalytic and nonautocatalytic routes, and not on the formal (and constant!) kinetic order of the autocatalyst used in the given rate equation. To support further this statement here, we also present our results comparing the simulations performed by eqs. 3–5 with that of eqs. 1–2 using p=1/2 in Figure 2.

As seen even though in case of eqs. 1–2 the rate coefficients belonging to the direct and autocatalytic pathways are smaller than in case of rate determining steps of the Landolt‐type systems (see: eqs. 3–4) the autocatalyst appears suddenly after a much shorter induction period in the previous case. Consequently, the strength of autocatalytic pathway has to be characterized rather by the parametrization than by the formal kinetic order of the autocatalyst.

The most important advantage of the kinetic model (eqs. 3–5) presented here is that (1) it simply does not require any artificial impurities to be introduced for interpreting the autocatalytic feature, (2) it is based on a law of mass action type kinetic model which is a straightforward possibility making a credit for chemical processes to proceed in reality even at prebiotic conditions.

### 2.2 Simple Law of Mass Action Type Autocatalytic Mechanism for ‘Superexponential Growth’

As it was mentioned ‘superexponential growth’ – when the kinetic order of the autocatalyst is higher than one – may also appear in polymerization models making it possible to enhance the autocatalytic feature of a given system.^21^ The term ‘supercatalysis’ was first introduced by Nagypál and Epstein more than 30 years ago,^34^ when the thiosulfate–chlorite reaction was thoroughly studied. It was shown that in one of the key subsystems of the parent reaction, namely in the tetrathionate–chlorite system, the formal kinetic order of the autocatalyst (hydrogen ion) is at least two, but at some certain conditions it may even grow further to three. Later, it was demonstrated that the system is also autocatalytic with respect to hypochlorous acid and chloride ion,^35^, ^36^ thus three autocatalysts with having different formal kinetic orders are involved in the kinetic model. Furthermore, it was also demonstrated that the formal kinetic order of hydrogen ion is also two not only in the direct reaction but even in the HOCl‐catalyzed pathway.^37^ Thus higher order of autocatalysis may easily appear in real chemical systems as well.

Let us now extend eqs. 3–5 by a reversible step with some modifications indicated in Table 1.

**Table 1:** A simple law of mass action type kinetic model for interpreting supercatalysis.

No.	Step	Rate equation
(1)	A+B→C	*v_I_*=*k_I_*[A][B]
(2)	B+C↔BC	*v_II_*=*k_II_*[B][C]
		*v*_*−II*_=*k*_*−II*_[BC]
(3)	BC+C→D	*v_III_*=*k_III_*[BC][C]
(4)	A+D→3C	*v_IV_*=*k_IV_*[A][D]

**Figure 3:**
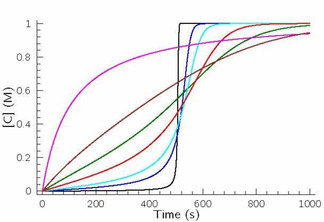
Concentration–time profiles of the autocatalyst at constant $k_{I}k_{II}k_{IV}/k_{III}$ =10^−5^ M^−3^ s^−2^ value ($k_{II}/k_{III}=0.01M^{-1}$ was considered in a rapidly established equilibrium having individual rate coefficients of $k_{II}=10^{6}$ M^−1^ s^−1^ and $k_{III}=10^{8}$ s^−1^, respectively) when varying the ratio of *k_IV_* and *k_I_*. [A]_0_=[B]_0_=1.0 M, [C]_0_=0 M and *k_V_*=10^7^ M^−1^s^−1^ was fixed. (A) *k_IV_/k_I_*=10^7^ (black), 10^5^ (blue), 10^3^ (green), 2.5×10^4^ (cyan), 4000 (red), 250 (brown), 10 (magenta).

Figure 3 indicates the results of the simulations. It can easily be seen that the ratio of the rate coefficients of the autocatalytic (No. (2)–(4) in Table 1) and the nonautocatalytic route (No. (1) in Table 1) determines whether the characteristic sigmoid shape of the concentration–time profiles is manifested or not. At high values the S‐shape is easily seen, though the profiles are clearly asymmetric differing significantly from those of found in cases of first‐order autocatalysis (see: previous subsection). In the present case, the sequence of reactions No. (2)–(3) in Table 1 represents the overall stoichiometry of

B+2C→D,

with a rate equation of

$$\upsilon {}_{III}=k{}_{III}\left[\mathrm{BC}\right]\left[C\right]=k{}^{'}{}_{III}\left[B\right]\left[C\right]{}^{2}$$

where $k_{III}^{'}$ =$k_{III}k_{II}/k_{-II}$ , because No. (2) in Table 1 is established rapidly, thus the equality of [BC]=$k_{II}/k_{-II}$ [B][C] has to be fulfilled. It straightforwardly shows that the formal kinetic order of the autocatalyst is 2, thus one has to envisage second‐order autocatalysis or supercatalysis. It should also be highlighted that appearance of supercatalysis may be identified in practice by measuring asymmetric sigmoidal concentration–time profiles, though it does not necessarily mean that an asymmetric sigmoidal curve has to uniquely belong to supercatalytic feature (see: Figure 2).

### 2.3 Hypercycles in Batch Conditions

Let us consider the simplest hypercycle consisting of two autocatalytic reactions connected by mutual catalysis of their autocatalyst. In order to fulfill this criterion let us first supplement eqs. 3–5 by the following sequence of reactions:

(6) $$X+Y\overset{k_{4}}{\to }Z,\;v_{4}=k_{4}\left[X\right]\left[Y\right]$$

(7) $$Y+Z\overset{k_{5}}{\to }W,\;v_{5}=k_{5}\left[Y\right]\left[Z\right]$$

(8) $$X+W\overset{k_{6}}{\to }2Z,\;v_{6}=k_{6}\left[X\right]\left[W\right]$$

So far these reactions are completely independent systems, thus sequence of eqs. 3–5 is linked to that of eqs. 6–8 by the following consideration resulting in mutual catalysis of C and Z autocatalysts:

(9) $$A+Z\overset{k_{7}}{\to }\mathrm{AZ},\;v_{7}=k_{7}\left[A\right]\left[Z\right]$$

(10) $$\mathrm{AZ}+B\overset{k_{8}}{\to }C+Z,\;v_{8}=k_{8}\left[\mathrm{AZ}\right]\left[B\right]$$

(11) $$X+C\overset{k_{9}}{\to }\mathrm{XC},\;v_{9}=k_{9}\left[X\right]\left[C\right]$$

(12) $$\mathrm{XC}+Y\overset{k_{10}}{\to }Z+C,\;v_{10}=k_{10}\left[\mathrm{XC}\right]\left[Y\right]$$

As it is seen the reaction between A and B is catalyzed by Z, which is the autocatalyst of reaction of X and Y. Similarly, C – the autocatalyst of the previous system – catalyzes the reaction between X and Y. Consequently, this system thus forms the simplest hypercycle.

**Figure 4:**
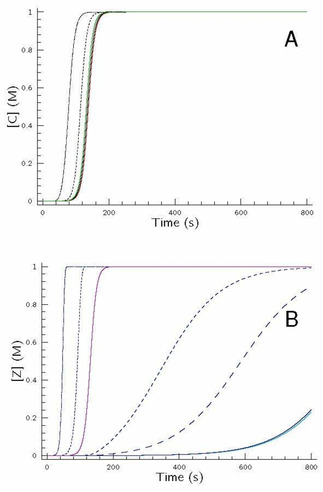
Concentration–time profiles of species C (upper part denoted by A) and Z (lower part denoted by B) in the simplest hypercycle when varying the rate coefficients (*k*_9_) of the rate determining step of the catalytic route of the cycle involving X,Y and Z. All the other rate constants were fixed during the simulations as *k*_1_=10^−7^ M^−1^ s^−1^, *k*_2_=10^−1^ M^−1^ s^−1^; *k*_6_=10^−6^ M^−1^ s^−1^; *k*_7_=10^−2^ M^−1^ s^−1^ and *k*_3_=*k*_5_=k_8_=*k*_10_=10^7^ M^−1^ s^−1^. The solid lines indicated by red and cyan colors provide the concentration–time profiles of C and Z, respectively considering that *k*_4_ =*k*_9_=0 thus the cycles are independent of each other. Rest of the simulations was performed by using *k*_4_=3×10^−3^ M^−1^ s^−1^ values. The solid lines indicated by black and blue colors represent the concentration–time profiles of C (black) and Z (blue), respectively. *k*_9_/M^−1^ s^−1^=3×10^−7^ (solid lines); 10^−4^ (long‐dashed line); 10^−3^ (medium‐dashed line); 1 (short‐dashed lines); 10 (dotted lines). Note that in case of the black curves at lower *k*_9_<10^−3^ M^−1^ s^−1^ simulated curves of C are basically overlapping each other. To make the difference a bit more visible when *k*_9_=0.1 M^−1^ s^−1^ is used the corresponding concentration–time curves of C and Z are represented by green and magenta solid lines. They almost overlap each other as well.

Let us also suppose that $k_{3}\gg k_{2}$ , $k_{3}\gg k_{1}$ , $k_{6}\gg k_{4}$ , $k_{6}\gg k_{5}$ , $k_{8}\gg k_{7}$ and $k_{10}\gg k_{9}$ inequalities are fulfilled. Such prerequisites mean that the concentration–time curves of products display sigmoid profiles (see: Figure 4). Evidently, in batch conditions both reactions (A+B→C and X+Y→Z) will reach complete conversion, but the induction periods may vary significantly. Figure 4 displays concentration–time profiles of the autocatalysts, when the contribution of the catalytic pathways (*k*_4_ and *k*_9_) is varied. The autocatalytic pathway dominates the reaction (exponential growth) in case of reactants A and B, meanwhile the nonautocatalytic pathway successfully competes with the autocatalytic route (parabolic) in case of reactants X and Y.

From this figure it is clear if the cross‐catalytic route (eqs. 4 and 9) becomes more and more pronounced then the induction period decreases steadily. This decrement may even appear such an extent that the conversion of Z in the otherwise sluggish ‘parabolic growth’ overcomes that of C in the ‘exponential growth’. It is therefore evident that there must be such a situation when the concentration–time profiles of both species completely overlap each other. In this case although the autocatalytic route is not so pronounced in case of the cycle containing species X,Y and Z, but the cross‐catalytic influence of C (the autocatalyst of the other cycle) is so effective to compensate the more sluggish autocatalytic route resulting in a faster appearance of species Z. Therefore, it may straightforwardly be concluded that in a hypercycle at batch conditions the autocatalytic route having a very efficient support from the crosscatalytic pathway may easily lead to such a situation where the otherwise more sluggish autocatalytic cycle would have a much stronger autocatalytic route. In other words a more efficient autocatalytic route does not necessarily mean that the cycle involving this sequence of reactions is always completed within the shortest period of time at batch conditions. It is also easy to see that this conclusion may be extended to such hypercycles where the number of autocatalytic cycles connected via mutual catalysis exceeds the simplest number, two. To our belief this argument also confirms the misuse of terms ‘exponential’ and ‘parabolic’ characterizing the fundamental features of autocatalytic processes, when these terms are considered to reflect just to the differences of the kinetic orders of the autocatalyst. It seems to be quite reasonable to believe that these attributes are straightforward consequences of parametrization, hence the relationship between rate coefficients of the autocatalytic and the nonautocatalytic pathways determines whether behavior of the given sluggish or strongly autocatalytic system satisfies the criterion of being ‘parabolic’ or ‘exponential’.

### 2.4 Hypercycles in Flow Systems

A more exciting approach is when hypercycles are considered to be driven under flow conditions when constant input of the reagents and output of the reactants and products are provided. These circumstances are mimicking the persistence of natural, inanimate processes in the prehistoric Earth, when feedstock (the reagents) is constantly supplied. Again, the simplest possible hypercycle (containing two autocatalytic loops by mutually catalyzing the cycles with each other′s autocatalyst) is employed to reveal the response of the system at flow conditions. Three different options have to be separated according to the behavior of two autocatalytic loops performed in batch system. The first option is when one of the autocatalyst in the hypercycle appear substantially shorter period of time than the other one at batch conditions (option A), the second possibility is when the concentration–time profiles of both autocatalyst under batch condition are overlapping each other (option B) and finally, the third possibility is when the concentration–time profiles of the competing autocatalysts cross each other at approximately a halfway conversion (option C). In all these calculations we considered that there is no difference at all in the initial concentrations of the reagents A, B, X and Y, as well as in their feeding concentrations at flow condition to provide equal wining chance for the products C and Z in the evolutionary race.

**Figure 5:**
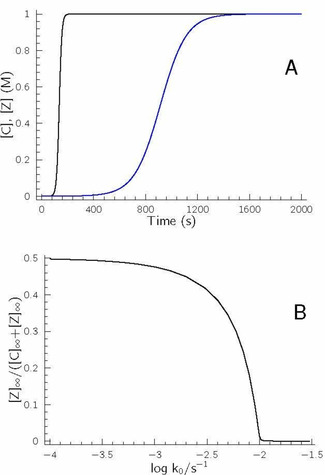
A: Concentration–time profiles of the autocatalyst C (black) and Z (blue) in the simplest hypercycle at batch condition. The rate coefficients of eqs. 1–10 can be found in the caption of Figure 4. The colors of solid lines match with the corresponding ones found in Figure 4. B: Change of the steady‐state concentration of the autocatalyst Z over that of the total autocatalyst concentration at flow conditions as a function of log*k*_0_.

Let us discuss option A first. Figure 5A indicates the concentration–time profiles of the autocatalyst under batch conditions, while Figure 5B displays the concentration ratio of the autocatalysts when steady‐state condition is reached in a flow system.

Figure 5B clearly suggests that no difference in the steady‐state concentration of the autocatalyst may be observed when the rate of inflow and outflow is too small, because in case of long residence time both autocatalytic routes spend enough time in the pool to reach high conversion for providing equal amount of autocatalyst. Of course, this situation [C]_∞_=[Z]_∞_ is a straightforward consequence of their same feedstock supply concentration as well as of the same initial concentrations of reactants. If, however, the residence time decreases, the [Z]_∞_/([C]_∞_+[Z]_∞_) ratio is gradually shifted from 0.5 to zero meaning that one of the autocatalytic routes in the hypercycle vanishes to operate anymore. One can easily visualize that under the conditions of our example it happens around log(*k*_0_)=−2, meaning that the residence time (*τ*) is 100 s. This time point coincides with the one needed to ignite the autocatalytic formation of C at batch conditions as seen in Figure 5A. It also provides an additional support that the most efficient autocatalytic route finally takes its advantage to eventually accumulate its autocatalyst over the other autocatalysts participating in the given hypercycle at a suitable flow condition. Consequently, in such cases, it means that in an evolutionary competition among all replicators the one having the most efficient autocatalytic route finally finds its way to spread out in the pool overwhelmingly over the rest of competitors. In other words products of sluggish autocatalytic routes are retarded by simply washing them out from the pool to prevent the accumulation of autocatalyst such an extent to ignite their own autocatalytic production.

**Figure 6:**
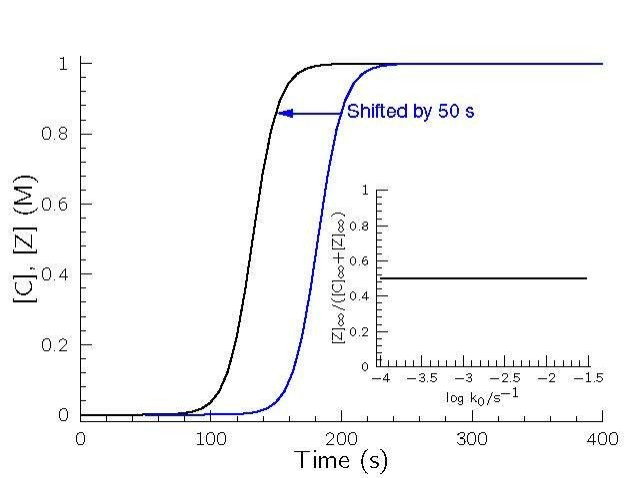
Concentration–time profiles of the autocatalyst C and Z in the simplest hypercycle at batch condition. The rate coefficients of eqs. 1–10 can be found in the caption of Figure 4. The only exception is *k*_9_ that is set to be 0.093 M^−1^ s^−1^. Inset: Unchanged steady‐state concentration ratio of the autocatalyst C and Z at flow condition as a function of log*k*_0_.

It is also interesting to examine what happens when the condition of option B is fulfilled, namely no notable difference may be encountered between the concentration–time profiles of the competitive autocatalyst in the simplest hypercycle. This situation may easily be induced by different parameter sets as seen in Figure 6. One may easily expect that the change of flow condition in this case does not have any effect to select between the competitive autocatalysts. As Figure 6 indicates, indeed, the concentration ratio of the competitive autocatalyst remains unchanged thus when although the kinetic parameter set of the governing models differs from each other significantly, both subsystems produce the same concentration–time profile of its corresponding autocatalyst, and the change in flow rate is not able to pave the way for one of the competitors to exist overwhelmingly over the other one. It also supports that parametrization may be considered as a key issue in characterizing autocatalytic processes.

**Figure 7:**
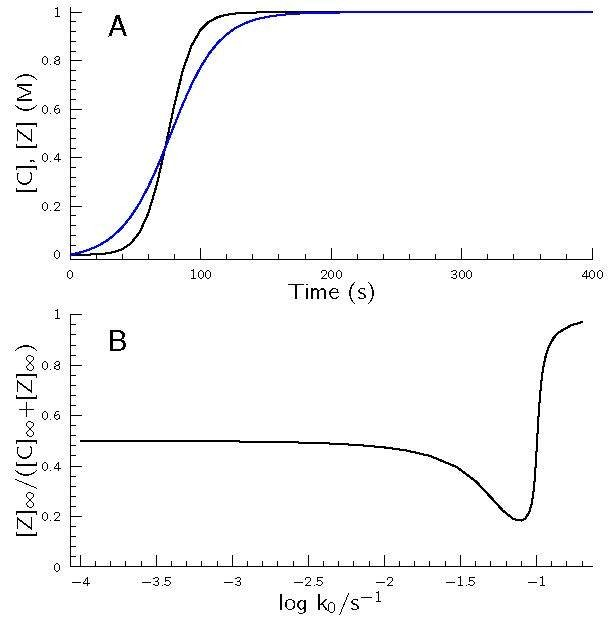
A: Concentration–time profiles of the autocatalyst C (black) and Z (blue) in the simplest hypercycle at batch condition. The rate coefficients of eqs. 1–10 can be found in the caption of Figure 3. The exceptions are *k*_6_=0.002 M^−1^ s^−1^, *k*_7_=0.02 M^−1^ s^−1^ and *k*_9_=0.03 M^−1^ s^−1^. B: Undershoot‐overshoot steady‐state concentration ratio of the autocatalyst C and Z at flow condition as a function of log*k*_0_.

Last, but not least it is also worthwhile to examine option C when at batch conditions the sigmoidal shape curves of the two competitive autocatalysts cross each other at nearly 50 % conversion. Such a situation is demonstrated in Figure 7A.

As seen at lower *k*_0_ values, i. e., at longer residence times both cycles have enough time to reach total conversion, consequently, the amount of both autocatalysts at steady‐state conditions agrees perfectly. If the residence time becomes shorter then there is not enough time for reagents X and Y to spend in the reactor reaching high conversion, but the other route (involving A and B) is still rapid enough to produce autocatalyst C with high conversion, therefore this species is found to be in significant excess over species Z. As it is seen in Figure 7B, around log(*k*_0_)=−1.1 more than 80 % of the total amount of autocatalyst exists in form of Species C. Further increase in *k*_0_, however, straightforwardly means that the steady‐state concentration of species C falls below that of species Z (see: Figure 7A at shorter reaction times) therefore the concentration ratio of the aforementioned autocatalysts is reversed, thus species Z finally appears at an overwhelming excess in the reactor, even though this route is not so effective as the other one in kinetic sense.

These calculations revealed a conclusion of great importance in mimicking the biological evolution modeled by hypercycles. Here it is clearly shown that not only the kinetic parameters of the governing routes of the given hypercycle have decisive role whether which of the autocatalysts may accumulate in overwhelming excess over the other one in the reactor but also the flow rate may select among the autocatalysts – involved in the hypercycle – to be favored at the given circumstances. It means that not always the strongest autocatalytic route in a batch condition may find its way eventually to spread out overwhelmingly in a flow reactor, but the one that is capable of adapting most efficiently the corresponding parametric circumstances meaning that natural selection may conveniently be modeled by hypercycles.

## 3 Conclusions

One of the most exciting and challenging question in chemical biology is certainly unraveling the unbelievably long puzzle‐road led to the appearance of life from inanimate matters to self‐replicatory complex living systems and undoubtedly it still provides plenty of unresolved clues to be investigated by the curious human nature. It is out of question that adequate explanation of this attractive story must await for further unpredictably long follow‐up studies. It looks to be evident that in the early ages after the birth of Earth under prebiotic conditions limited number of inanimate matters may have been found. Consequently, it means that limited number of possible direct law‐of‐mass‐action‐type reactions may lead to the formation of a template molecule that may later assist in its own formation in an accelerating manner. Such a simple autocatalytic mechanism is examined here in detail that may as well be extended to give rise even more complex autocatalytic feature such as super‐exponential growth and hypercycles. As a result, it seems to be an inherent characteristics of these complex systems that in an evolutionary race emergence of overwhelming excess of the wining template molecule among the other participants mainly depends on those external factors (like concentration fluctuations, temperature change, variable inflow and outflow rate, etc.) that decisively characterize the concentration–time profiles of the competitive species. In other words it means that adaptation to the ever‐changing open condition in our ancient planet was really a key factor in the evolutionary race and this feature can easily be modeled by hypercycles. This possibility seems to be implicitly involved in the above mentioned positive feedback processes when flow conditions are applied. As it is seen natural selection among inanimate template molecules may therefore be explained adequately though an important question is still to be answered: How did the first very primitive living organisms appear in our ancient Earth from inanimate matters?

## Conflict of interest

The authors declare no conflict of interest.
